# Mn-euvering manganese: the role of transporter gene family members in manganese uptake and mobilization in plants

**DOI:** 10.3389/fpls.2014.00106

**Published:** 2014-04-01

**Authors:** Amanda L. Socha, Mary Lou Guerinot

**Affiliations:** Department of Biological Sciences, Dartmouth CollegeHanover, NH, USA

**Keywords:** manganese, metal transport, Arabidopsis, rice, synchrotron x-ray fluorescence

## Abstract

Manganese (Mn), an essential trace element, is important for plant health. In plants, Mn serves as a cofactor in essential processes such as photosynthesis, lipid biosynthesis and oxidative stress. Mn deficient plants exhibit decreased growth and yield and are more susceptible to pathogens and damage at freezing temperatures. Mn deficiency is most prominent on alkaline soils with approximately one third of the world's soils being too alkaline for optimal crop production. Despite the importance of Mn in plant development, relatively little is known about how it traffics between plant tissues and into and out of organelles. Several gene transporter families have been implicated in Mn transport in plants. These transporter families include NRAMP (natural resistance associated macrophage protein), YSL (yellow stripe-like), ZIP (zinc regulated transporter/iron-regulated transporter [ZRT/IRT1]-related protein), CAX (cation exchanger), CCX (calcium cation exchangers), CDF/MTP (cation diffusion facilitator/metal tolerance protein), P-type ATPases and VIT (vacuolar iron transporter). A combination of techniques including mutant analysis and Synchrotron X-ray Fluorescence Spectroscopy can assist in identifying essential transporters of Mn. Such knowledge would vastly improve our understanding of plant Mn homeostasis.

## Introduction

With an increasing human population, there is a growing demand for improvements in crop production. Mineral nutrients such as iron (Fe), copper (Cu), and zinc (Zn) are essential for both plants and animals, but maintaining optimal micronutrient levels presents challenges in both the plant and animal kingdoms. To combat human nutrient deficiency, efforts are being made to increase the bioavailability of nutrients in the edible portions of plants (Schroeder et al., 2013). Another concern is contamination of the soil with non-essential metal(oid)s such as arsenic (As), lead (Pb), cadmium (Cd), and mercury (Hg) (Salt et al., 1998). These elements are detrimental to both plants (through direct uptake from the soil) and humans (through consumption of contaminated plant products). One strategy for remediating soil contaminated with metals is using hyperaccumulator plants, which can tolerate and store remarkably high levels of metals in the aerial portion of the plant (Kramer, 2010). Mn is an example of an element that is both required in humans but can potentially be toxic. In excess, Mn can induce neurological symptoms that resemble Parkinson's disease (Martinez-Finley et al., 2013). Therefore, reducing human exposure to high levels of Mn is a major worldwide health concern. By better understanding the molecular mechanisms, specifically which transport proteins allow plants to take up and store metals, steps can be taken toward improved crop growth and the engineering of biofortified plants for improved human nutrition.

**Figure 1:**
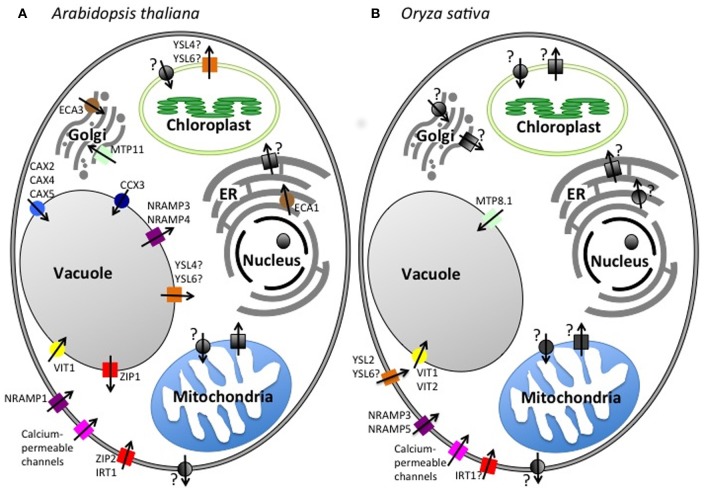
**Subcellular localization of putative Mn transporters.** A diagram of a plant cell showing the Mn transport pathways in **(A)** *A. thaliana* and **(B)** *O. Sativa.* Squares, import into the cytosol; Circles, export out of the cytosol; Gray, unknown; Red, ZIP family; Magenta, Calcium-permeable channels; Orange, YSL family; Light blue, CAX family; Dark Blue, CCX family; Yellow, VIT family; Purple, NRAMP family; Brown, P2A-Type ATPase family; Green, CDF/MTP family.

**Figure 2:**
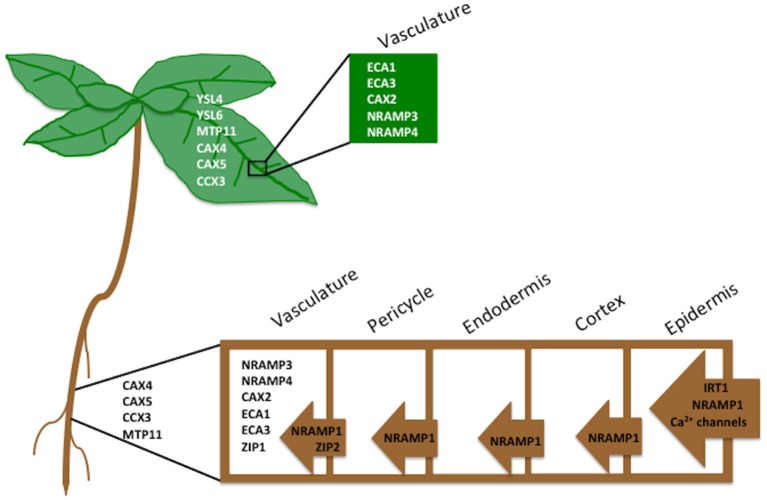
**Tissue localization of Mn transporters.** The probable role of transporter family members in translocating Mn from the soil into the aerial portion of the plant in *A. thaliana*. The transporters listed to the left of the cells are not yet localized to a specific tissue.

This review will focus on the micronutrient Mn. Despite its importance in cellular processes, such as photosynthesis and protecting cells against reactive oxygen species (ROS), little is known about transporters essential for Mn uptake and storage in the cell. We describe common and emerging techniques for assaying Mn localization and accumulation within the plant. In addition, we detail the protein families implicated in Mn transport. Particular emphasis will be placed on the model plant species *Arabidopsis thaliana* and *Oryza sativa* (rice), on which the majority of characterization work has been carried out. The transporter families will be organized based on their putative function of transporting Mn into or out of the cytoplasm. Table 1 summarizes the transporters discussed in this review. The subcellular localization (in *A. thaliana* and rice) and tissue localization (in *A. thaliana*) of the transporters is shown in Figures 1 and 2, respectively.

**Table 1:** **Putative Mn transporters referenced in the text**.

**Transporter**	**Tissue expression**	**Subcellular localization**	**Transcript response to Mn deficiency**	**Transcript response to Mn toxicity**	**Other proposed substrates**	**References**
**NRAMP FAMILY**						
AtNRAMP1	Root (all tissues) > shoot	PM	Up in root	- -	Fe, Cd	Curie et al., 2000; Thomine et al., 2000; Cailliatte et al., 2010
AtNRAMP3	Shoot and root vasculature, developing seed	VM	None	- -	Fe, Cd	Thomine et al., 2000
AtNRAMP4	Shoot vasculature > root vasculature, developing seed	VM	None	- -	Fe, Zn, Cd	Thomine et al., 2000
OsNRAMP3	Nodes > root and leaf vasculature, panicle, husk, flower	PM	None	None (PTM)		Yamaji et al., 2013
OsNRAMP5	Root exodermis and endodermis	PM	None	–	Cd	Ishimaru et al., 2012; Sasaki et al., 2012
DMTI	Root, leaf, stems	PM/peri-bacteroid membrane	- -	- -	Fe, Zn, Cu	Kaiser et al., 2003
LeNRAMP1	Root	VM	- -	- -		Bereczky et al., 2003
LeNRAMP3	Root > shoot	VM	- -	- -		Bereczky et al., 2003
**YSL FAMILY**						
AtYSL4	Shoot, silique, root, flower, developing seed	VM/EM/CM	None	- -	Ni, Fe^**^	Conte et al., 2013; Divol et al., 2013
AtYSL6	Shoot, flower, silique, developing seed	VM/EM/CM	None	- -	Ni, Fe^**^	Conte et al., 2013; Divol et al., 2013
OsYSL2	Leaf and leaf sheath phloem, root phloem, developing seed	PM	None	- -	Fe	Koike et al., 2004
OsYSL6	Root, shoot	Un-determined	None	- -	- -	Sasaki et al., 2011
ZmYS1	Root epidermis, leaf mesophyll	PM	- -	- -	Fe, Zn, Cu, Ni, Cd	Roberts et al., 2004; Schaaf et al., 2004; Ueno et al., 2009
**ZIP FAMILY**						
AtIRT1	Root epidermis, flower	PM	None	- -	Fe, Zn, Cu, Cd, Co	Eide et al., 1996; Korshunova et al., 1999; Vert et al., 2002
HvIRT1	Root	PM, ER	None	- -	Fe, Zn	Pedas et al., 2008
PsIRT1	Root	PM	- -	- -	Fe, Zn, Cd	Cohen et al., 2004
LeIRT1	Root, flowers	- -	- -	- -	Fe, Zn, Cd	Eckhardt et al., 2001
LeIRT2	Root	- -	- -	- -	Fe, Zn, Cd	Eckhardt et al., 2001
AtZIP1	Root vasculature > shoot vasculature	VM	Up in shoot	- -	Zn	Milner et al., 2013
AtZIP2	Root vasculature > shoot vasculature	PM	Down in shoot	- -	Zn	Milner et al., 2013
AtZIP5	Root > shoot	- -	- -	- -		Milner et al., 2013
AtZIP6	Root > shoot	- -	- -	- -		Milner et al., 2013
AtZIP7	Shoot > root	- -	- -	- -	Zn, Fe	Milner et al., 2013
AtZIP9	Root, shoot	- -	- -	- -		Milner et al., 2013
MtZIP4	Leaf, root^*^	MM (predicted)	Down in leaf	- -		Lopez-Millan et al., 2004
MtZIP7	Leaf	PM (predicted)	None	- -		Lopez-Millan et al., 2004
**CAX FAMILY**						
AtCAX2	Root, shoot and flower vasculature, fruit, stem	VM	- -	None	Ca, Cd, Zn	Hirschi et al., 2000; Schaaf et al., 2002; Shigaki et al., 2003; Pittman et al., 2004; Edmond et al., 2009
AtCAX4	Root, leaf, stem, flower, silique	VM	- -	Up	Cd	Cheng et al., 2002
AtCAX5	Root, stem, fruit, flower, leaf	VM	- -	Up		Edmond et al., 2009
OsCAX1a	Root, shoot, flower	- -	- -	- -	Ca	Kamiya and Maeshima, 2004; Kamiya et al., 2005
OsCAX3	Root, shoot, flower	- -	- -	- -	Ca	Kamiya and Maeshima, 2004; Kamiya et al., 2005
LeCAX2	Leaf, fruit	- -	- -	- -	Ca	Edmond et al., 2009
HvCAX2	Root, shoot, seed	- -	- -	None	Ca	Edmond et al., 2009
**CCX FAMILY**						
AtCCX3	Flowers, stem, leaf, root	VM, EM	- -	Up in root and flowers	Na, K	Morris et al., 2008
**CDF/MTP FAMILY**						
AtMTP11	Leaf hydathodes, guard cells, root tip	Golgi/PVC	- -	None		Delhaize et al., 2007; Peiter et al., 2007
OsMTP8.1	Shoot	VM	None	Up in shoot		Chen et al., 2013
ShMTP8	- -	Internal organelle	- -	- -	- -	Delhaize et al., 2003
PtMTP11.1	- -	GLC	- -	- -		Peiter et al., 2007
PtMTP11.2	- -	GLC	- -	- -		Peiter et al., 2007
BmMTP10	Root, shoot	Golgi	- -	Up in root and shoot		Erbasol et al., 2013
BmMTP11	Root, shoot	Golgi	- -	None		Erbasol et al., 2013
**P-type ATPase FAMILY**						
AtECA1	Root vasculature, flower, leaf vasculature, stem, silique	ER	- -	- -	Ca, Zn, Ni	Wu et al., 2002
AtECA3	Root vasculature and tip, Leaf vasculature, hydathodes, guard cells, flower, stem, silique	Golgi/EM	None	- -	Ca	Mills et al., 2008
LeLCA1	- -	- -	- -	- -	Ca	Johnson et al., 2009
**VIT/CCC1-LIKE FAMILY**						
AtVIT1	Developing seed, vasculature	VM	- -	- -	Fe	Kim et al., 2006
OsVIT1	Leaf > root, stem, panicle, embryo	VM	- -	- -	Fe, Zn	Zhang et al., 2012
OsVIT2	Leaf > root, stem, panicle, embryo	VM	- -	- -	Fe, Zn	Zhang et al., 2012

* *Only during Zn deficiency*.

** Not observed by Conte et al. (2013). At, Arabidopsis thaliana; Hv, Hordeum vulgare; Ps, Pisum sativum; Le, Lycopersicon esculentum (Solanum lycopersicum); Mt, truncatula; Os, Oryza sativa; Zm, Zea mays; Bm, Beta vulgaris; PM, Plasma Membrane; ER, Endoplasmic Reticulum; VM, Vacuolar Membrane; MM, Mitochondrial Membrane; EM, Endomembrane Compartment; PVC, Pre-Vacuolar Compartment; GLC, Golgi-Like Compartment; PTM, Post-Translational Modification; - -, not tested.

## Symptoms of Mn deficiency and Mn toxicity

Mn is a micronutrient element required for normal plant growth and development. It is essential for most photosynthetic organisms as a component of the oxygen-evolving complex in photosystem II (PSII), which catalyzes the water-splitting reaction to produce oxygen and provides electrons for the photosynthetic electron transport chain (Goussias et al., 2002; Nickelsen and Rengstl, 2013). Mn is also required for multiple steps in carbohydrate, lipid and lignin biosynthesis in plants (Marschner, 2012). Mn acts as a direct cofactor of a variety of enzymes (~35 in plants), which include decarboxylases of the TCA cycle, RNA polymerases and numerous glycosyl transferases. In some enzymes, other metals can replace Mn as an enzymatic cofactor (Hebbern et al., 2009). Typically magnesium (Mg) replaces Mn because it is 50–100 times more abundant in the cell. Conversely, when Mn is in excess, it can replace Mg, which can have detrimental effects on the cellular processes in which Mg is involved. There are some enzymes that specifically require Mn such as those involved in cellular redox reactions. Mn is an indispensable component of Mn superoxide dismutase (MnSOD), a principal antioxidant enzyme of the mitochondria. A recent study analyzing the effect of Mn deficiency on Chlamydomonas showed that MnSOD activity decreases before that of PSII (Allen et al., 2007). This finding suggests intracellular regulation of Mn to support PSII function in the chloroplast in preference to MnSOD function in the mitochondria.

Plant uptake of Mn is a function of the Mn oxidation state in the soil. While Mn can exist in a range of oxidation states (Mn^1+^, Mn^2+^, Mn^3+^, Mn^4+^, Mn^6+^, and Mn^7+^), the most commonly found forms in biological systems are Mn^2+^, Mn^3+^ and Mn^4+^with Mn^4+^ being the least stable (Marschner, 2012). The most soluble species in soil is the divalent cation, Mn^2+^, which is also the form of Mn that is most efficiently accumulated in plants (Marschner, 2012). Soil pH is a major determinant of Mn oxidation state in soil. At neutral or higher pH, Mn^3+^ and Mn^4+^ predominate and insoluble Mn oxides will form (Rengel, 2000; Marschner, 2012). Mn solubility is also influenced by microorganisms, which can either reduce or oxidize Mn, thereby affecting its availability to the plant (Lovley et al., 2011; Geszvain et al., 2012).

Despite its necessity, Mn is required in relatively small amounts (~20–40 milligrams per kilogram of dry weight in most crop species) (He et al., 2005; Jiang, 2006; Marschner, 2012). However, it is one of the most prevalent trace element deficiencies seen in cereals including wheat and barley (Jiang, 2006). Fertilizers containing Mn sulphate can be added to the soil, however this is costly and the added Mn can be oxidized making it unavailable for plant acquisition. A more cost-effective and eco-friendly alternative is to preferentially breed plants that are able to thrive in low Mn soil. There has been a significant effort to identify crop species that are considered Mn efficient and can tolerate growth on low Mn, either by storing more Mn or by having an increased ability to absorb Mn from the soil (Jiang, 2006; Pedas et al., 2008). The genetic basis of Mn efficiency is not well understood.

Mn-deficient plants exhibit inhibited growth and decreased biomass (Marschner, 2012). Interveinal chlorosis due to decreases in net photosynthesis and chlorophyll content are common. This is most likely because the Mn-complex is needed to stabilize the PSII reaction center protein, D1 (Krieger et al., 1998; Allen et al., 2007; Yanykin et al., 2010). Mn-deficient plants are also characterized by tissue necrosis due to a decrease in MnSOD levels and an increase in oxygen free radicals (Allen et al., 2007; Marschner, 2012). Because Mn is a cofactor in the biosynthesis of cinnamic acid and its polymerization into lignin, decreased lignin concentration is especially prominent in the roots of Mn deficient plants (Marschner, 2012; Salvador et al., 2013). This is believed to be a contributing factor to an increased susceptibility to damage by freezing temperatures and root-infecting pathogens (Marschner, 2012). Mn deficiency can result from multiple factors including high concentrations of other minerals in the soil [i.e., Fe, Mg, calcium (Ca), phosphorus (P)] that can interfere with Mn absorption as well as soil alkalinity (pH >7.5) (Lynch and St. Clair, 2004; Marschner, 2012). Highly calcareous soils and soils found in arid and semi-arid regions are described as too alkaline for vegetative growth (Lynch and St. Clair, 2004).

Mn toxicity can also be detrimental to plants. Toxicity can occur in poorly drained acidic soils (pH < 5.5), where there is a predominance of Mn^2+^ (Lynch and St. Clair, 2004). Excess Mn can also prevent the uptake and translocation of other essential elements such as Ca, Mg, Fe, and P, presumably due to the similarity in ionic radius or binding strength for ligands (Marschner, 2012; Millaleo et al., 2013). In addition, toxicity can inhibit PSII and increase the accumulation of oxidized Mn and oxidized phenolic compounds in the leaf apoplast (Fecht-Christoffers et al., 2003; Marschner, 2012). As a result, common symptoms of Mn toxicity include interveinal chlorosis and tissue necrosis, which manifests as brown spots on mature leaves of plants, ultimately resulting in reduced plant biomass (Marschner, 2012). There are multiple mechanisms proposed to combat the deleterious effects of excess Mn. For example, plants can sequester Mn in the apoplast or vacuole (Horst and Maier, 1999; Hirschi et al., 2000; Schaaf et al., 2002). In fact, ectopic expression of vacuolar Mn transporters can increase the plant's tolerance to excess Mn (Delhaize et al., 2003). Free Mn ions can be chelated in metabolically inactive Mn^2+^-organic acid complexes (Horst and Maier, 1999; Pittman, 2005; Fernando et al., 2010). In addition, Mn^2+^ can be mobilized into the endoplasmic reticulum (ER), thereby reducing cytoplasmic Mn (Wu et al., 2002).

## Techniques for studying metal transport and accumulation in plants

The range of metals transported by a particular transport protein can be determined by expressing the gene in a simpler model, usually yeast. Some of the yeast strains commonly used to test metal transport capabilities are *smf1*, *fet3fet4*, *ctr1*, and *zrt1zrt2*, which are unable to transport Mn, Fe, Cu, and Zn, respectively, across the plasma membrane (Dancis et al., 1994; Dix et al., 1994; Supek et al., 1996; Zhao and Eide, 1996a,b). Specific metal transport by a protein of interest is indicated by rescued growth of these yeast strains in a low Mn, Fe, Cu or Zn media or in the presence of a divalent metal chelator. For example, the *smf1* mutant strain is unable to grow on media containing the divalent cation chelator EGTA. Another commonly used yeast strain to assay metal transport is *pmr1.* The P_2_-type Ca-ATPase, PMR1 (plasma membrane ATPase related 1) pumps both Ca^2+^ and Mn^2+^ in the Golgi for detoxification purposes or for use as a cofactor for Golgi-localized proteins (Rudolph et al., 1989; Durr et al., 1998). When PMR1 is defective, yeast are more sensitive to high concentrations of Mn^2+^ (Durr et al., 1998). Therefore, complementation of *pmr1* yeast with a Mn efflux transporter should restore growth when Mn in the media is high. Indirect studies using yeast, such as competition assays, are sometimes used to determine if a transporter has broad specificity (examples can be found in Grotz et al., 1998; Kaiser et al., 2003). However, further studies are necessary to confirm biological function.

Xenopus oocytes, immature eggs of an aquatic frog, are used to study the physiological function of a transporter. Electrophysiological measurements can be recorded in this system as well as uptake of radioactively labeled metals like ^54^Mn. This system also allows for the addition of any potential metal chelators necessary for Mn translocation across the membrane. While it is not clear whether Mn-specific metallochaperones exist in plants, Mn can complex with nicotianamine (NA), phytosiderophores (PS), phytate and organic acids (Koike et al., 2004; Haydon and Cobbett, 2007; Fernando et al., 2010).

Mn content in plant tissues or in yeast expressing a plant transporter is measured to demonstrate difference in Mn transport efficiency. To date, the most accurate and sensitive method to measure metal content in a sample is Inductively Coupled Plasma Mass Spectrometry (ICP-MS) (Baxter et al., 2008; Donner et al., 2012). However, because ICP-MS requires the total digestion of a sample, it does not collect spatial information about an element *in vivo*. For some metals, ion specific fluorophores exist, which, in conjunction with confocal microscopy, can image the subcellular localization of a metal (Burdette et al., 2001; Miller et al., 2006, 2008; Yoon et al., 2007), but there is currently no Mn-specific fluorescent probe. Therefore, other techniques are needed to spatially resolve Mn localization in a cell.

Quantitative *in vivo* cryo-scanning electron microscopy (SEM)/energy dispersive X-ray analysis (EDAX) has been used to provide detailed electron micrographs of tissue from hyperaccumulator plants along with energy dispersive X-ray spectra from regions of interest (Fernando et al., 2006b).The samples are prepared by rapidly freezing them in liquid nitrogen, which preserves the metal location during processing and microbeam exposure (Fernando et al., 2013). Further analysis using Particle-Induced X-ray Emission induced by a focused ion beam (μ PIXE) was used to confirm the cryo-SEM/EDAX results (Fernando et al., 2006a). Synchrotron X-Ray Fluorescence (SXRF) technology is a method used to localize metals *in vivo* at resolutions down to 250 nm. For a review on how SXRF can be used to study gene function see Punshon et al. (2013). It is important to recognize that no one method stands alone in determining the role of a protein in Mn translocation and multiple methods must be used in parallel.

## Manganese localization *in planta*

Intracellular Mn is found in multiple locations in the cell including the chloroplast, cell wall, mitochondria and Golgi apparatus (Pittman, 2005). Mn is also located in the vacuole, an organelle that is critical for cellular metal homeostasis, where it serves as an intracellular sink when metals are in excess and as a source when metals are limited (Pittman, 2005; Fernando et al., 2006a; Lanquar et al., 2010). Much of the Mn imaging in plant tissues has been performed on Mn hyperaccumulators, due to their unique metabolism and ease of imaging Mn-enriched tissues (Leitenmaier and Kupper, 2013). To date, there are around 22 Mn hyperaccumulators identified around the world (Fernando et al., 2013). The majority of hyperaccumulator plants' primary sequestration sites are in non-photosynthetic tissues such as trichomes (leaf hairs) and epidermal tissue. However, Mn hyperaccumulators can sequester excess Mn in photosynthetic tissues as well as non-photosynthetic tissues. In previous studies of five different Mn hyperaccumulators, excess foliar Mn was concentrated in the double layer of chloroplast containing palisade mesophyll cells in the leaf (Fernando et al., 2006a,b). In contrast, the Mn hyperaccumulator, *Maytenus founieri*, exhibited increased Mn accumulation in the non-photosynthetic leaf epidermal tissues, which is reminiscent of other metal hyperaccumulators such as the Zn/Cd hyperaccumulator *A. halleri* (Fernando et al., 2008).

**Figure 3:**
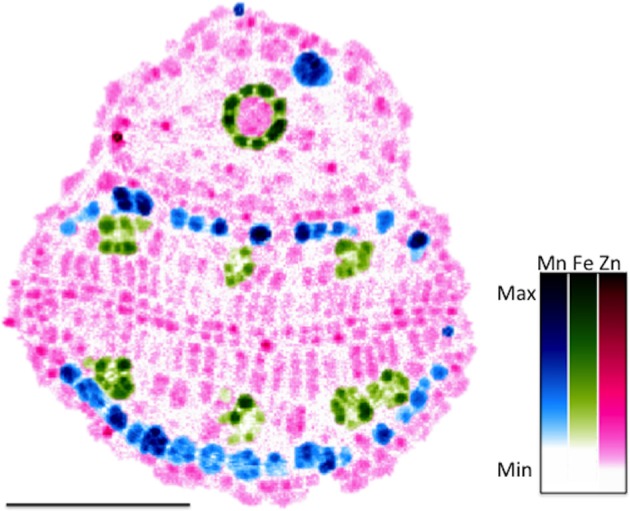
**Elemental distribution of Mn, Zn and Fe in *A. thaliana* seeds collected using Synchrotron X-Ray Florescence.** Two dimensional map of a cross-section of an *A. thaliana* seed collected at 1 micron resolution at the Advanced Photon Source. Mn (blue), Zn (magenta), and Fe (green) are shown.

SXRF can be used to spatially localize and quantify Mn in *A. thaliana* seeds at a resolution high enough to resolve metals at a subcellular level (Donner et al., 2012). Recent studies using μPIXE confirmed SXRF data that Mn is localized to spongy mesophyll on the abaxial side of the embryonic leaf (Kim et al., 2006; Schnell Ramos et al., 2013) (Figure 3). Donner et al. (2012) pointed out that, due to its requirement for photosynthesis, Mn needs to relocate to the palisade mesophyll cells during germination.

## Manganese transporters

Studying the metal transport mechanisms of other species can greatly assist our understanding of how plants take up and distribute Mn. While Mn transport is relatively understudied in plants, it is well understood in bacterial and yeast systems (Jkubovics and Jenkinson, 2001; Culotta et al., 2005; Papp-Wallace and Maguire, 2006; Reddi et al., 2009). In the yeast, *Saccharaomyces cerevisiae*, Mn is transported into the cell through the high affinity Mn^2+^ transporter SMF1 of the NRAMP family (Supek et al., 1996) and the high affinity phosphate transporter, PHO84 (Jensen et al., 2003). PHO84-type phosphate transporters prefer neutral metal-phosphate complexes; therefore the substrate is presumed to be MnHPO_4_ (Jensen et al., 2003). The photosynthetic alga *Chlamydomonas* also encodes an NRAMP protein that is predicted to be involved in high affinity Mn^2+^ influx (Allen et al., 2007). Photosynthetic autotrophs such as cyanobacteria require Mn for PSII assembly and a gene product with sequence similarity to an ABC (ATP binding cassette)-type transporter is implicated in Mn uptake in this organism (Bartsevich and Pakrasi, 1995).

There are few Mn-only transporters identified in plants. One possible explanation is that Mn shares many of the same transporters as other divalent cations such as Fe. One study found that it might not be possible to uncouple Mn and Fe transport in one high affinity Fe transporter after systematically introducing point mutations into the metal binding domain of IRT1 (Rogers et al., 2000). In fact, the majority of transporters implicated in Mn translocation have broad specificity for several divalent cations including Fe, Zn, Cu, Cd, Ca, Co (cobalt), Ni (nickel). The transporter families discussed below are organized based on transport of Mn into or out of the cytosol.

### Transport into the cytosol

#### NRAMP family

NRAMP1 was first characterized in mice as a gene involved in resistance to intracellular pathogens (Nevo and Nelson, 2006). Like many of the transporter families implicated in Mn transport, the NRAMPs transport a broad range of metals and can transport Mn^2+^ as well as Fe^2+^, Zn^2+^, Cd^2+^, Cu^2+^, Ni^2+^, Co^2+^, and Al^3+^ (Nevo and Nelson, 2006; Xia et al., 2010). The plant NRAMP proteins are predicted to be ion transporters based on sequence and predicted structural homology to the NRAMPs characterized in organisms such as humans and yeast. NRAMPs have between 10 and 12 transmembrane domains and a consensus transport sequence between transmembrane domains 8 and 9 that is shared with other ion channels and transporters (Cellier et al., 1995).

NRAMP proteins have been characterized in a number of plant species including *Solanum lycopersicum*, *Glycine max*, *Malas baccata*, *Thlaspi japonicum* and hyperaccumulators *A. halleri* and *Thlaspi caerulescens* (Bereczky et al., 2003; Kaiser et al., 2003; Mizuno et al., 2005; Xiao et al., 2008; Oomen et al., 2009). There are 6 and 7 NRAMP transporter proteins in *A. thaliana* and *Oryza sativa*, respectively. The first NRAMP genes cloned from plants were from rice in 1997 (Belouchi et al., 1997). However, not all are functionally characterized (Bennetzen, 2002; Nevo and Nelson, 2006).

Functional characterization of AtNRAMP1, AtNRAMP3, and AtNRAMP4 was first reported in 2000 (Thomine et al., 2000). AtNRAMP1 is believed to be a high affinity Mn transporter in the root due to the transcriptional upregulation of *AtNRAMP1* in the root under Mn deficiency and its PM localization (Cailliatte et al., 2010). In addition, *AtNRAMP1* expression increases in Fe deficiency and the protein complements yeast defective in Fe or Mn transport (Curie et al., 2000). Studies show that Arabidopsis *Atnramp1* knockout lines are more susceptible to Mn deficiency, accumulate less Mn in the shoot when grown without Mn and have decreased Mn in the root when grown under Mn replete conditions (Cailliatte et al., 2010). Plants overexpressing *AtNRAMP1* are more resistant to Mn deficiency. Elemental images of Mn in *Atnramp1–1* mutant seeds shows wild type distribution of Mn, therefore it is unlikely that these genes are necessary for Mn loading into the embryonic cotyledons (Donner et al., 2012).

AtNRAMP3 and AtNRAMP4 are also able to complement yeast deficient in either Mn or Fe uptake. *Atnramp3–1* mutants show increased Mn in the roots of plants grown in Fe limited conditions while Mn accumulation decreased when *AtNRAMP3* is expressed in the cell under control of the strong 35S promoter (Thomine et al., 2003). This phenotype is possibly due to the vacuolar localization of this transporter. Expression data and transcriptional fusions to the promoters of *AtNRAMP3* and *AtNRAMP4* show that the transporters are expressed in the root stele and the vasculature of leaves and cotyledons and are highly upregulated during Fe deficiency (Thomine et al., 2000, 2003; Lanquar et al., 2005). AtNRAMP4 was identified as part of the vacuolar proteome in *A. thaliana* mesophyll cells (Carter et al., 2004). Translational fusions of both AtNRAMP3 and AtNRAMP4 proteins show that they are localized to the vacuolar membrane; therefore they are thought to be important for metal remobilization from the vacuole, *in planta* (Thomine et al., 2003).

AtNRAMP3 and AtNRAMP4 transporters are functionally redundant; only *Atnramp3nramp4* double mutants exhibit a strong phenotype in response to metal deficiency. Both AtNRAMP3 and AtNRAMP4 are considered necessary for Fe remobilization during early germination due to their high expression levels at this growth stage (Lanquar et al., 2005). Lanquar et al. (2010) demonstrated that AtNRAMP3 and AtNRAMP4 are necessary for Mn export from the vacuole in the mesophyll cells of adult plant leaves. Double mutants have increased Mn accumulation in vacuoles. Under Mn deficiency, double mutants exhibit stunted growth and chlorosis when compared to wild type. Expression of either AtNRAMP3 or AtNRAMP4 in the double mutant can restore growth in Mn deficiency. Interestingly, it has been shown in other photosynthetic organisms that MnSOD activity decreases before there is a loss of function PSII in response to Mn deficiency (Allen et al., 2007). However, in Arabidopsis *Atnramp3nramp4* double mutants, the level of active PSII decreases while there is no loss of MnSOD activity (Lanquar et al., 2010). These results suggest intra-organellar crosstalk between the chloroplast and vacuole that mediate proper Mn distribution in the cell.

To date, it is not known whether AtNRAMP2, AtNRAMP5 or AtNRAMP6 are important for Mn homeostasis.

Of the 7 NRAMPs in rice, only 4 have been functionally characterized. *O. sativa* plants utilize OsNRAMP3 to respond to environmental changes in Mn availability. *OsNRAMP3* is constitutively expressed in the node of rice, which connects the vasculature of the root to aerial tissues of the plant (including the leaves, panicles and stems) (Yamaji et al., 2013). Under Mn deficiency, the PM localized OsNRAMP3 transports Mn from the transpiration stream in the xylem of enlarged vascular bundles to the younger tissues and panicles to meet minimal growth requirements. In contrast, when Mn is in excess, OsNRAMP3 is internalized in vesicles and rapidly degraded. Then, Mn is preferentially loaded into the older leaves, which are directly connected to the xylem-enlarged vascular bundles, thereby protecting developing tissue from Mn toxicity. This study demonstrates the importance of post-translational regulation in response to environmental nutrient availability.

*OsNRAMP5*, on the other hand, does not respond to varying levels of environmental Mn, although gene expression increases slightly in the roots when plants are under Fe or Zn deficiency (Sasaki et al., 2012). Expression is seen in the mature zones of the root, more specifically at the PM of the exodermal and endodermal layers. Knockdown and RNAi lines accumulate less Mn in the roots and shoots and growth is stunted in response to Mn deficiency (Ishimaru et al., 2012; Sasaki et al., 2012). This phenotype is specific to Mn and not to other metal deficiencies tested, such as Fe-deficiency (Sasaki et al., 2012). Supplementing the plant with excess Mn does not rescue the growth defect. Therefore, it is likely that OsNRAMP5 is essential for Mn uptake from the soil in rice.

#### YSL family

The YSL transporters belong to a poorly characterized family of oligopeptide transporters (OPTs), which can transport amino acid-containing compounds and their derivatives (Yen et al., 2001). They are not found in all kingdoms, rather they are only found in plants, bacteria, fungi and archaea (Yen et al., 2001). The family is named after maize YS1 (ZmYS1), which accumulates in the root PM when Fe is scarce (Von Wiren et al., 1994; Roberts et al., 2004; Schaaf et al., 2004; Ueno et al., 2009). The characteristic interveinal chlorosis in the leaf of *ys1* knockdown mutants prompted the name “yellow stripe.” Transport studies in both yeast and oocytes suggest that it can transport metals such as Mn, Zn, Cu, Ni, Cd as well as Fe (Schaaf et al., 2004). Therefore, it is reasonable to hypothesize that YSLs can transport Mn in other plant species.

YSL proteins mediate cellular uptake of metals complexed to non-proteinogenic amino acids: PS or its biosynthetic precursor NA (Yen et al., 2001). There is evidence that PS and NA are ligands for Mn (Schaaf et al., 2004; Haydon and Cobbett, 2007). Graminaceous plants (grasses) that include many crop species, use PS and NA for metal uptake and translocation, respectively (Palmer and Guerinot, 2009). PS is not produced by non-graminaceous plants (such as *A. thaliana*). Therefore, YSLs in non-grasses most likely use NA for intercellular and intracellular metal transport (Bashir et al., 2011).

There are eight predicted *YSL* genes in *A. thaliana* based on sequence similarity to YS1 (Curie et al., 2001). Knocking out a single *AtYSL* gene does not produce a strong visible phenotype (Didonato et al., 2004; Waters et al., 2006; Curie et al., 2009; Conte et al., 2013; Divol et al., 2013). However, it was discovered that if crosses are made between *Atysl* knockouts whose genes are in the same AtYSL subclass, a visible phenotype is observed. Examples include *Atysl1ysl3, Atysl4ysl6*, and *Atysl5ysl7* (Curie et al., 2009; Conte et al., 2013; Divol et al., 2013). Proteomics studies of the Arabidopsis vacuole predicted AtYSL4 and AtYSL6 to be vacuolar-localized (Jaquinod et al., 2007). Evidence shows that they are either located at the vacuole/endocompartments (shown via translational fusions) or the chloroplast (shown via immunofluorescence) (Conte et al., 2013; Divol et al., 2013). Data suggests that these proteins may be effluxers of heavy metals from an internal cellular compartment. Conte et al. (2013) observed that in comparison to wild type and single mutants, the double mutant is more resistant to prolonged growth on excess Mn. This phenotype was not reported by Divol et al. (2013). Thus far, the physiological role of AtYSL4 and AtYSL6 in Mn homeostasis remains to be clarified.

In rice, there are 18 putative *YSL* genes (Koike et al., 2004; Conte et al., 2013). OsYSL2 and OsYSL6 are implicated in Mn homeostasis. Electrophysiological and localization studies suggest the OsYSL2 is involved in lateral movement of Mn^2+^-NA complexes via the phloem and loading into developing seeds (Koike et al., 2004). OsYSL6 is required for Mn detoxification when soil Mn is in excess (Sasaki et al., 2011). OsYSL6 is expressed constitutively in all cells of the roots and the shoots, particularly in senescing leaves. GFP-fusions of the protein disrupt localization, but due to successful complementation of the *smf1* yeast mutant, it is hypothesized to localize to the PM. When expressed in yeast, OsYSL6 translocates Mn^2+^-NA. In *Osysl6* knockouts, plants exhibit symptoms of toxicity and excess Mn accumulates in the apoplast of the roots and shoots. When Mn^2+^ accumulates in the apoplast, it is oxidized into Mn^3+^, which in turn will oxidize proteins and lipids (Fecht-Christoffers et al., 2003). Therefore, it is probable that when plants are exposed to high levels of Mn, OsYSL6 translocates Mn from the apoplast to the symplast where it is properly sequestered.

#### ZIP family

The *ZIP* gene family members are known to transport a broad range of metals including Fe^2+^, Zn^2+^, Cd^2+^, Co^2+^, and Mn^2+^ in both eukaryotes and prokaryotes (Korshunova et al., 1999; Guerinot, 2000). Structurally, they are composed of eight transmembrane domains with the N- and C- termini facing outside the PM. Their length and amino acid composition varies within the cytoplasmic variable region between transmembrane domains 3 and 4. The variable region contains a conserved histidine motif that is believed to be important for metal binding and point mutations of inter-membrane domains can alter metal selectivity (Guerinot, 2000; Rogers et al., 2000).

The ZIP family of metal transporters is named for its founding members in both *S. cerevisae* and *A. thaliana*, respectively (Grotz et al., 1998). Of the 16 ZIP transporters in Arabidopsis, few have been functionally characterized (Maser et al., 2001; Rampey et al., 2013). IRT1 (Iron Regulated Transporter 1) is a high affinity Fe transporter that also has low affinity for other metals including Mn (Eide et al., 1996; Korshunova et al., 1999; Vert et al., 2002; Yang et al., 2008). It is upregulated in Fe deficient roots. In addition, exogenous Mn cannot rescue the chlorotic phenotype of *irt1* mutants, which only grow in the presence of excess Fe (Vert et al., 2002). However, *irt1* mutant plants accumulate less Mn in their tissues during Fe deficiency, suggesting that IRT1 is a major pathway for Mn^2+^ uptake when Fe is scarce (Connolly et al., 2002). Because Mn uptake is still required for the plant during Fe replete conditions, there is likely another PM membrane localized transporter on the root epidermis that can transport Mn. Interestingly, under Mn^2+^ deficiency, IRT1 transcript levels decrease and Fe transport increases as evidenced by high Fe concentrations in the plant (Yang et al., 2008).

A more recent study found that in addition to AtIRT1, six *A. thaliana* ZIPs could restore growth to the Mn uptake defective *smf1* mutant: AtZIP1, AtZIP2, AtZIP5, AtZIP6, AtZIP7, and AtZIP9 (Milner et al., 2013). AtZIP3, AtZIP4, AtZIP10, AtZIP11, AtZIP12 did not. *AtZIP8* is a pseudogene in Arabidopsis and this did not complement any of the yeast strains used in the study. Under normal conditions, AtZIP1, AtZIP2, and AtZIP6 are more highly expressed in the root while AtZIP7 transcript is more highly concentrated in the shoot. *AtZIP5* and *AtZIP9* transcripts are not very abundant and can be found in the root and shoot. The physiological roles of AtZIP6, AtZIP7, and AtZIP9 in Mn homeostasis *in planta* remain to be explored.

*AtZIP1* is most highly expressed in the root stele and is localized to the vacuole (Milner et al., 2013). AtZIP1 is believed to remobilize Mn from vacuoles into the cytoplasm to allow for their translocation to the shoot. This function is supported by the phenotype of *Atzip1* T-DNA insertion lines, which are more sensitive to low Mn and accumulate more Mn in the root. AtZIP2 is proposed to supply Mn to the aerial tissues in the plant via the root vasculature and not through direct uptake from the soil based on it localization to the PM in the root stele. In addition, mutant plants lacking functional AtZIP2 are more tolerant to Mn toxicity and less tolerant to Mn-deficiency, suggesting their importance in Mn translocation to the shoot for sequestration of excess Mn or normal plant health. However, it is unlikely that AtZIP2 is the primary transporter of Mn in the root because gene expression decreases during Mn deficiency.

To date, the literature does not detail any OsZIPs that are able to transport Mn. However, *OsZIP5* transcript was slightly induced in the root upon Mn deficiency (Lee et al., 2010). Yet, it was not able to rescue *smf1* mutants and is therefore unlikely to be a direct transporter of Mn. ZIPs are described in other plant species, aside from Arabidopsis, that transport Mn. These species include *Solanum lycopersicum*, *Pisum sativa*, *Hordeum vulgare*, and *Medicago truncatula* (Eckhardt et al., 2001; Bereczky et al., 2003; Cohen et al., 2004; Lopez-Millan et al., 2004; Pedas et al., 2008). Tomato LeIRT1 and LeIRT2 along with PsIRT1, HvIRT1, MtZIP4, and MtZIP7 can restore growth to *smf1* mutants in Mn limited media. In barley, Mn efficiency correlates with increased expression of HvIRT1, an OsIRT1 ortholog, in roots (Pedas et al., 2008).

#### Calcium-permeable channels

Calcium-permeable channels located in the PM of the root cells are a Ca^2+^ influx pathway that may be permeable to Mn^2+^ (White et al., 2002). Initial studies of these channels used biochemical and electrophysiological experiments to characterize their role in catalyzing cation influx because the genes encoding these channels had not been identified. A subset of cation channels that may be permeable to Mn^2+^ (as well as a broad range of other cations) are found in the apical PM of Arabidopsis root hairs (Very and Davies, 2000), protoplasts from the endodermis, cortex and root elongation zone and the epidermis of a developing root tip (Kiegle et al., 2000). They are also identified in the stele of maize roots (White et al., 2002). In addition, *LCT1*, a gene in wheat roots that is potentially a Ca^2+^-permeable channel, was cloned and heterologously expressed in a yeast mutant where the transport of Ca^2+^ is blocked by the addition of Mn^2+^ to the media (Clemens et al., 1998). There is further evidence based on competition assays that Ca^2+^-permeable channels transport Mn^2+^ in *A. thaliana* root hair tips (Wymer et al., 1997) and maize roots (Marshall et al., 1994).

### Export from the cytosol

#### CAX family

The CAX proteins are one of five transporter families that constitute the Ca^2+^/cation antiporters (CaCA) superfamily (Shigaki and Hirschi, 2006; Emery et al., 2012). Although the CAX family members were originally identified as Ca^2+^ transporters, further study revealed their ability to transport a wide array of ions, hence their name modification from “calcium exchanger” to “cation exchanger.” Typically, the CAXs contain 11 transmembrane domains and are found in plants, fungi and bacteria and in lower vertebrates. They facilitate the redistribution of cations across a membrane using electrochemical energy generated by a proton pump in order to maintain optimal ionic concentrations in the cell.

There are six CAX proteins in *A. thaliana*, which are split into two distinct phylogenetic groups: AtCAX1, AtCAX3, and AtCAX4 are part of the Type IA subgroup and AtCAX2, AtCAX5, and AtCAX6 are part of the Type 1B subgroup (Shigaki et al., 2006). The biological significance of the subgroups is unclear. In plants, the CAXs mediate efflux of ions into the vacuole. AtCAX2 contains a three-amino acid Mn^2+^ binding domain (Cys-Ala-Phe) between transmembrane domain 4 and transmembrane domain 5 (Shigaki et al., 2003). Expression of AtCAX2 in yeast can confer tolerance to Mn^2+^ toxicity (Schaaf et al., 2002; Shigaki et al., 2003). Furthermore, ectopic expression of AtCAX2 in tobacco (*Nicotiana tabacum*) can mediate vacuolar sequestration of Mn and confer resistance to high Mn stress (Hirschi et al., 2000), indicating a Mn transport function. In *A. thaliana*, *AtCAX2* is expressed at low levels in all tissues and does not respond to changes in Mn availability (Hirschi et al., 2000). However, Arabidopsis *Atcax2* mutants accumulate significantly less Mn in the vacuole compared to wild type (Pittman et al., 2004). The *Atcax2* knockout mutants display no obvious phenotype, even under Mn stress, and some Mn does continue to accumulate in the vacuole, suggesting that there are other vacuolar transporters that can compensate for the absence of AtCAX2 (Pittman et al., 2004). Candidates include AtCAX4 and AtCAX5 along with the AtCCX3 and AtVIT1 transporters, which will be discussed below.

AtCAX4 and AtCAX5 are likely involved in Mn^2+^/H^+^ antiport activity. Like AtCAX2, AtCAX4, and AtCAX5 are located at the vacuolar membrane and are constitutively expressed at low levels in all tissues (Cheng et al., 2002; Edmond et al., 2009). AtCAX5 is most highly expressed in the stem and root and AtCAX4 is more highly expressed in the roots (determined via qRT-PCR) and plays a role in root growth under ion stress, such as Mn^2+^ toxicity (Edmond et al., 2009; Mei et al., 2009). Interestingly, AtCAX4 and AtCAX5 RNA levels increase when plants are exposed to high Mn^2+^(Cheng et al., 2002; Edmond et al., 2009; Mei et al., 2009). AtCAX4 increases Mn stress tolerance when expressed in tobacco and AtCAX5 can rescue Mn-sensitive yeast, indicating their ability to transport Mn (Korenkov et al., 2007; Edmond et al., 2009).

Thus far, single *Atcax* mutants do not exhibit strong phenotypes, even when exposed to Mn stress. This is possibly due to functional redundancy of the AtCAX proteins. A subset of double mutants in Arabidopsis are reported in the literature: *Atcax1cax3*, *Atcax2cax3*, and *Atcax1cax2* (Connorton et al., 2012; Punshon et al., 2012). AtCAX1 appears to be important for determining the phenotype of a double mutant plant. A study by Connorton et al. (2012), found that *Atcax2* and *Atcax2cax3* mutants are both more sensitive to high Mn than wild type. However, the *Atcax1cax2* double mutant does not display any signs of Mn toxicity, suggesting that by deleting *Atcax1* from the *Atcax2* mutants, Mn is once again able to accumulate in the vacuole. The phenotype of the *Atcax1cax2* mutant is consistent with the phenotype of the plants lacking a functional *AtCAX1* gene, which are also more tolerant to Mn toxicity. Therefore, it will be important to understand the apparent cross-talk that occurs between the AtCAX proteins, which confers their Mn transport capabilities.

There are five CAX proteins in the rice genome, all of which have been cloned and their transport specificities assessed in yeast (Kamiya et al., 2005). OsCAX1a and OsCAX3 confer Mn tolerance in yeast, therefore they are potentially Mn^2+^/H^+^ exchangers *in planta* (Kamiya and Maeshima, 2004; Kamiya et al., 2005). It is important to note that CAX proteins can also transport Mn in other plant species. Examples include HvCAX2 in barley and LeCAX2 in tomato (Edmond et al., 2009). Future work is needed to confirm that they are also localized to the vacuole *in planta*, where they mediate Mn tolerance.

#### CCX family

CCXs are one of five families of transporters, (along with the CAXs) which make up the CaCA superfamily (Shigaki et al., 2006; Emery et al., 2012). There are five CCX proteins (CCX1-5), which were previously identified as CAX7-11. They were reclassified due to their higher sequence homology to the mammalian PM K^+^-dependent Na^+^/Ca^2+^ exchangers (NCXs). Expression of AtCCX3 in yeast rescues mutants defective in either PM or vacuolar import of Mn^2+^ (Morris et al., 2008). However, GFP and HA tagged versions of the protein support localization to intracellular compartments. Wild type yeast expressing AtCCX3 had nearly double the Mn concentration when compared to yeast expressing the control vector. Also, when ectopically expressed in tobacco, plant Mn^2+^ concentration significantly increased as leaves matured, which led to tissue necrosis. This phenotype is likely due to the defects in ion homeostasis in the plant resulting in increased ROS as evidenced by higher oxidation of proteins compared to controls. In plants, treatment with Mn induces *AtCCX3* expression in roots and flowers (Morris et al., 2008). Like *Atcax* single mutants, there are no apparent growth defects in Arabidopsis *Atccx3* mutant plants, which could be due to functional redundancy. Interestingly, AtCCX3 can transport both monovalent and divalent cations. However, the only divalent cation that can be transported by AtCCX3 is Mn^2+^. Whether other AtCCXs can translocate Mn^2+^ remains to be studied.

#### CDF/MTP family

The CDF family, also known as MTP, is ubiquitous among all kingdoms of life (Maser et al., 2001; Montanini et al., 2007). The CDFs act as proton antiporters, which efflux metals such as Zn^2+^, Fe^2+^, Co^2+^, Ni^2+^, Cd^2+^, and Mn^2+^ out of the cytoplasm or into subcellular compartments (Gustin et al., 2011). However, one CDF protein is reported to be responsible for Zn uptake into the cytoplasm (Cragg et al., 2002). The plant CDFs are clustered into three functional groups based on phylogenetic analysis: Zn-CDFs, Fe/Zn-CDFs, and Mn-CDFs (Montanini et al., 2007; Gustin et al., 2011). Metal selectivity of the proteins is inferred from metal transport activity (either confirmed or hypothesized) of the respective members of the three subgroups. Mn-CDFs also contain amino acid residues that may predict metal specificity. The CDFs typically have six transmembrane domains and Mn-CDFs contain the highly conserved consensus sequence DXXXD (where *X* = any amino acid) in transmembrane domains 2 and 4, which is not found in the Zn- or Fe/Zn-CDFs (Montanini et al., 2007). Studies show that even single point mutations within key structural sites can alter metal ion specificity of the MTPs. For example, single point mutations in AtMTP1 (Podar et al., 2012) and OsMTP1 (Menguer et al., 2013) allow Mn uptake, which is not observed with the wild type protein.

In plants, the CDFs are identified as MTPs due to their role in detoxification of heavy metals (Ricachenevsky et al., 2013). The first characterized Mn-CDF transporter was identified in the Mn hyperaccumulating tropical legume, *Stylosanthes hamata* (Delhaize et al., 2003). ShMTP8 (previously annotated ShMTP1) can confer Mn^2+^ tolerance when ectopically expressed in Arabidopsis or yeast by sequestering excess metal in the vacuole. Of the twelve identified CDFs in *A. thaliana*, there are four in the Mn-CDF subgroup, which are also highly similar to ShMTP8, suggesting similar function. These proteins are AtMTP8, AtMTP9, AtMTP10, AtMTP11. The Mn-CDFs are further divided into subgroup 8 (including AtMTP8 and ShMTP8) and subgroup 9 (including AtMTP9, AtMTP10, and AtMTP11). To date, AtMTP11 is the only functionally characterized Arabidopsis Mn-CDF. Yeast transformed with AtMTP11are more tolerant to Mn^2+^ (Delhaize et al., 2007; Peiter et al., 2007). In addition, increased Mn^2+^ dependent proton-transport activity was recorded in yeast microsomal vesicles prepared from yeast expressing AtMTP11 in comparison to vesicles prepared from the control yeast strain (Delhaize et al., 2007). These data further support the role of AtMTP11 as an Mn^2+^/H^+^ antiporter. Two independent studies localized AtMTP11 to the pre-vacuolar and Golgi-like compartments. Surprisingly, *AtMTP11* is most highly expressed in the leaf hydathodes and the root tip rather than in tissues that typically accumulate excess Mn such as the trichomes (Peiter et al., 2007). Hydathodes are involved in secretion of water containing salts and metals from the leaf. Therefore, Peiter et al. (2007) hypothesized that AtMTP11 is involved in vesicular trafficking and exocytosis of excess Mn at secretory tissues where Mn will be excreted rather than stored. This hypothesis is supported by the phenotype of both knockout mutants and plants overexpressing AtMTP11. Arabidopsis *Atmtp11* mutants are hypersensitive to high Mn while overexpression lines are hypertolerant to high Mn (Delhaize et al., 2007; Peiter et al., 2007). Also, the *Atmtp11* mutants accumulate more Mn in the shoot and root, presumably due to their inability to secrete Mn from tissues (Peiter et al., 2007).

In rice there are five Mn-CDFs: OsMTP8 and OsMTP8.1 from Group 8 and OsMTP9, OsMTP11, and OsMTP11.1 from group 9. Recently, OsMTP8.1 was identified in a screen of rice shoot cDNAs that conferenced Mn tolerance in yeast (Chen et al., 2013). *pmr1* yeast mutants expressing OsMTP8.1 also accumulated more Mn. In rice, OsMTP8.1 is mainly expressed in shoots under all conditions tested, and expression increases in response to excess Mn. *Osmtp8.1* knockdown and knockout mutants exhibited toxicity symptoms in the presence of elevated Mn and Mn accumulation in the roots and shoot compared to wild type was reduced. The protein is localized to the vacuole, resulting in the hypothesis that OsMTP8.1 is important for Mn detoxification by sequestering Mn into the vacuole in rice plants.

Two MTP11 orthologs (PtMTP11.1 and PtMTP11.2) in poplar are believed to have a similar function as AtMTP11 due to their localization to Golgi-like compartments and ability to complement Arabidopsis *Atmtp11* plants (Peiter et al., 2007). Another study in beets (*Beta vulgaris*) identified two genes, *BmMTP10* and *BmMTP11*, which were named after their orthologs in *A. thaliana*. Much like AtMTP11, these proteins are associated with the Golgi and are hypothesized to efflux excess Mn via the secretory pathway. The conservation of Mn-CDF function among multiple plant species supports the importance of these transporters in Mn detoxification and cellular homeostasis.

#### P-type ATPase family

The endomembrane system is essential for coordinating ion homeostasis in the cell. Several transporters that localize to the vacuolar membrane are described to facilitate metal uptake and release. However, there are also transporters in other endomembrane compartments that are equally important. Currently, there are two characterized proteins in Arabidopsis, AtECA1 (ER-type calcium ATPases) and AtECA3, which are localized to the ER and Golgi Apparatus, respectively (Liang et al., 1997; Wu et al., 2002; Li et al., 2008; Mills et al., 2008). AtECA1 and AtECA3 function as Mn^2+^ pumps that remove Mn^2+^ from the cytosol and deliver it into their respective endomembrane compartment.

The ECAs belong to the Ca^2+^-ATPase subfamily within the P-type ATPase superfamily of transporters, which use energy from ATP hydrolysis to catalyze the translocation of cations across membranes (Baxter et al., 2003; Huda et al., 2013). Plant Ca^2+^-ATPases are categorized into P_2A_ and P_2B_-types, both of which are generally described as Ca^2+^ pumps (Evans and Williams, 1998; Mills et al., 2008). There are four predicted P_2A_-type ECA proteins in *A. thaliana* (AtECA1-4) and three in rice (OsECA1-3) (Baxter et al., 2003). P_2A_-type ATPases show sequence homology to the sarcoplasmic/ER Ca^2+^-ATPases (SERCA type) found in mammals. SERCAs, PMCAs (plasma membrane Ca^2+^-ATPases) and SPCAs (secretory pathway Ca^2+^-ATPases) comprise three distinct subfamilies of P_2_-type Ca-ATPases in animal cells (Pittman et al., 1999; Mills et al., 2008). PMCAs are homologous to P_2B_-type PMCAs, while there are no known SPCAs in plants.

Both AtECA1 and AtECA3 are able to restore the growth of *pmr1* yeast when Mn is high (Wu et al., 2002; Li et al., 2008; Mills et al., 2008). Proteomics analysis of Arabidopsis organelles, translational fusions and co-localization with marker proteins supports the ER localization of AtECA1 as well as the Golgi localization of AtECA3 (Wu et al., 2002; Dunkley et al., 2006; Mills et al., 2008; Nikolovski et al., 2012). AtECA1 is expressed in all major organs (especially in the root and flower) where it is believed to play a major role in managing Mn toxicity in the cell (Wu et al., 2002). *Ateca1* mutants appear wild type when grown under standard conditions, yet are smaller and chlorotic in the presence of high Mn. Two different *Ateca3* mutants exhibit opposite phenotypes to Mn stress. *Ateca3-2* mutants are more susceptible to Mn deficiency (Mills et al., 2008). When grown without Mn, they display stunted growth and leaf chlorosis, which can be rescued by the addition of even trace amounts of Mn. The *Ateca3-4* mutant allele is more sensitive to Mn toxicity (Li et al., 2008). The phenotypes of plants carrying these alleles suggest that AtECA3 is important for pumping Mn into the Golgi for proper plant nutrition as well as detoxification via the Golgi compartments. Further investigation is needed to understand the variation between the alleles that uncouples the proposed dual function of the AtECA3 transporter.

There is no evidence that the Arabidopsis AtECA2 or AtECA4 transporters play a role in Mn homeostasis. In fact, *Ateca2* mutants appear similar to wild type when grown under high or low Mn (Mills et al., 2008). A tomato ECA, LCA1 (Lycopersicon esculentum Ca^2+^-ATPases), shows high amino acid sequence similarity to ECA2 (77%) in comparison to ECA1 and ECA3 (Pittman et al., 1999). LCA1 can also complement the *pmr1* yeast mutant, suggesting that it is also an endocompartment-localized Mn^2+^ pump (Johnson et al., 2009). Additional study of ECAs *in A. thaliana*, rice and other crop species is required to clarify their role in Mn homeostasis *in planta*.

#### VIT/CCC1-like family

CCC1 (Ca^2+^-sensitive cross complementer 1), which transports Fe and Mn into the vacuole in yeast (Li et al., 2001), has six orthologs in Arabidopsis referred to as CCC1-like (Rampey et al., 2006; Gollhofer et al., 2011). To date, AtVIT1 is the best characterized CCC1-like transporter and is likely involved in Mn uptake into the vacuole. As hypothesized, *ccc1* mutant yeast, when expressing AtVIT1, could accumulate Mn in the vacuoles (Kim et al., 2006). High-resolution SXRF images of *Atvit1* mutant seeds display highly localized Mn in the Arabidopsis embryo in a pattern identical to that seen in wild type. This is not wholly surprising considering the multitude of transporters that are implicated in Mn transport in the absence of AtVIT1. Therefore, although AtVIT1 is a potential route for Mn transport in planta, it does not appear to be essential for localization of Mn in the seed. The functional orthologs of AtVIT1 in rice (OsVIT1 and OsVIT2) are localized to the vacuole. They transport Mn, Fe and Zn in yeast, however physiological studies of mutants suggest that they are only Fe and Zn transporters *in planta* (Zhang et al., 2012).

## Conclusions and future perspectives

Mn, although essential for plant survival, can be toxic to plants. Over the past few decades, several transporter families have been identified that play a role in Mn homeostasis. Interestingly, many of the transporters that translocate Mn have broad specificity for other ions, particularly divalent cations. For example, transporters such as IRT1, originally described as an Fe transporter in the root, can also transport Mn into the plant. In addition, CAX family members along with ECA proteins were originally thought to transport Ca; it was only subsequent research that discovered their roles in Mn transport as well. Despite the advancements in identifying Mn transporters, there is still a lot unknown regarding the molecular mechanisms controlling Mn homeostasis in plants. Many Mn transporters are not differentially regulated at the transcriptional level in response to Mn stress. Further research can explore the possibility that many of the transporters are under post-transcriptional or post-translational control. The degradation of OsNRAMP3 in response to Mn toxicity in rice may be a common mechanism used by plants to tolerate stress conditions (Yamaji et al., 2013).

Further characterization of orthologs of Mn transporters found in *A. thaliana* in crop species is a necessary step toward agronomic advancement. Research involving identification of genes involved in Mn homeostasis can be exploited to generate plants that are able to thrive in suboptimal soil conditions which would increase crop production and guarantee food security. For example, if we breed plants with high Mn uptake efficiency in the root, we can grow plants in highly alkaline soils where plants usually succumb to Mn deficiency. In conjunction, developing plants with increased Mn storage capability can assist in either soil Mn detoxification or improved growth of plants in acidic soils.

### Conflict of interest statement

The authors declare that the research was conducted in the absence of any commercial or financial relationships that could be construed as a potential conflict of interest.
